# Modeling the effects of anomalous diffusion on synaptic plasticity

**DOI:** 10.1186/1471-2202-14-S1-P343

**Published:** 2013-07-08

**Authors:** Toma Marinov, Fidel Santamaria

**Affiliations:** 1 Department of Biology, The University of Texas at San Antonio, San Antonio, TX, 78249, USA

## 

The diffusion of cytosolic intracellular signals in spiny dendrites is anomalous due to spine trapping [1]. During anomalous diffusion the mean square displacement (MSD) of diffusing molecules follows a power law, MSD ~ t^α^, with α called the anomalous exponent. We have shown that α depends on the density and structure of spines and could be a general property of all spiny dendrites [2]. Anomalous diffusion affects the spatial spread and temporal concentration profiles of cytosolic molecules, thus potentially affecting the specificity and reliability of synaptic plasticity. Here we study the effect of anomalous diffusion on the spatial and the temporal distribution of signals involved in the expression of long term depression (LTD) in Purkinje cells (PCs). LTD depends on the PKC-MAPK positive feedback cascade. Increased [Ca2+] activates PKC, which in turn activates MAPK. Activated MAPK and [Ca2+] results in production of arachidonic acid which then activates PKC. The activated PKC either further activates MAPK or phosphorylates AMPARs, which are then removed from the synapse [3].

We use the fractional diffusion formulation of anomalous diffusion. In such a framework the diffusion-reaction equation for a given reactant is:

$$\frac{\partial^{\alpha }C_{R_{i}}}{\partial t^{\alpha }}=\gamma \nabla^{2}C_{R_{i}}+f\left(C_{R_{i}},C_{R_{j}}\right)$$

where α depends on the spine density along the dendrite, *γ*(t) is the generalized transport coefficient, C_Ri_(t) is the concentration of the reactant R_i _and f(C_Ri_, C_Rj_) defines the reaction terms of the specific biochemical reaction. Solving a system of coupled fractional diffusion-reaction equations for [Ca2+], PKC and MAPK is computationally expensive. To address this problem we recently developed a Fractional Integration Toolbox (FIT) [4].

**Figure 1:**
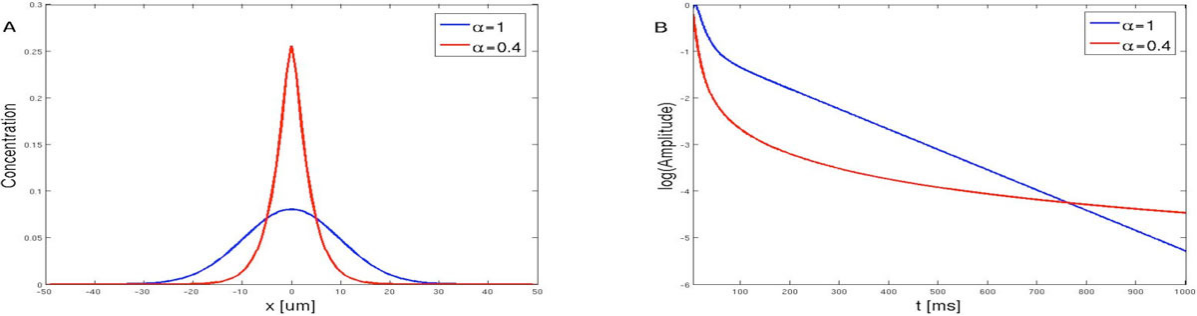
**Diffusion of PKC along a dendrite with no spines (α = 1) or high spine density (α = 0.4)**. (**A**) Spatial profile of PKC at t = 1 sec after release at × = 0, the anomalous diffusing PKC remains longer and with higher amplitude than the normally diffusing molecule. (**B**) The logarithmic transformation of the amplitude decay at × = 0 from the simulations in A show that the anomalous diffusing PKC stays activated longer than the normally diffusing PKC.

We have solved a simplified LTD model. In this model [Ca2+] does not undergo anomalous diffusion [1]. However, since PKC and MAPK are large proteins, they are susceptible to molecular trapping by spines resulting in anomalous diffusion. Our results show that in spiny dendrites (α < 1) the diffusion of either PKC or MAPK is slower than in the case of diffusion in spineless dendrites (α = 1) (Figure 1A). Under anomalous diffusion there is a longer activation of the PKC-MAPK positive feedback loop. Once activated, PKC and MAPK stay activated longer (Figure 1B), implying a lower [Ca2+] activation threshold. Thus, anomalous diffusion affects not only the spatial spread of molecules produced during LTD but also the activation threshold of the synaptic plasticity process.
